# Nutrient stoichiometry in winter wheat: Element concentration pattern reflects developmental stage and weather

**DOI:** 10.1038/srep35958

**Published:** 2016-10-24

**Authors:** M. Weih, F. Pourazari, G. Vico

**Affiliations:** 1 Department of Crop Production Ecology, Swedish University of Agricultural Sciences, Uppsala, Sweden

## Abstract

At least 16 nutrient elements are required by plants for growth and survival, but the factors affecting element concentration and their temporal evolution are poorly understood. The objective was to investigate i) element concentration pattern in winter wheat as affected by crop developmental stage and weather, and ii) whether, in the short term, element stoichiometry reflects the type of preceding crop. We assessed the temporal trajectories of element concentration pattern (N, P, K, Ca, Mg, S, Mn, Fe, Cu, Na, Zn) across the life cycle (from seed to seed) of winter wheat field-grown in cool-temperate Sweden during two years with contrasting weather and when cultivated in monoculture or after different non-wheat preceding crops. We found strong influence of developmental stage on concentration pattern, with the greatest deviation from grain concentrations found in plants at the start of stem elongation in spring. Inter-annual differences in weather affected stoichiometry, but no evidence was found for a short-term preceding–crop effect on element stoichiometry. Winter wheat element stoichiometry is similar in actively growing plant tissues and seeds. Nitrogen exerts a strong influence on the concentration pattern for all elements. Three groups of elements with concentrations changing in concert were identified.

Nutrients are important for plant growth and extensively supplied to managed terrestrial systems to enhance crop growth and yield. Despite at least 16 nutrient elements are required by plants^1^, most studies dealing with plant nutrition and stoichiometry focus on nitrogen (N) and phosphorus (P) because they are regarded as the most important in limiting plant growth and hence crop production^2^,^3^. Element concentration pattern and stoichiometry can vary depending on species or genotype, ontogeny and environment^4^,^5^,^6^. In general, plant growth rate increases approximately linearly with the concentrations of most nutrient elements in the plant tissues up to an optimum nutrient concentration beyond which a further increase in the element concentration is not accompanied by increased plant growth^5^. It is often assumed that changes in the variation in tissue element concentrations are closely mirrored by the changes in N concentration, provided N (and not other elements) is the most limiting factor for plant growth. Nevertheless, this common assumption in nutrient research has been rarely tested especially for agricultural crops.

Plant nutrition research in agricultural context frequently focuses on the harvested product, occasionally with the addition of a single vegetative crop stage (e.g. anthesis in cereals). The whole life cycle of the crop, i.e., “from seed to seed” is rarely investigated. Nutrient requirements, nutrient uptake rates and element stoichiometry vary across the life cycle of plants including agricultural crops^4^,^7^,^8^,^9^,^10^. Element stoichiometry in plants is affected partly by soil element availability in combination with uptake capacity of the plant independent of its growth rate, and partly by the element uptake of the plant largely driven by its growth rate^1^,^11^. Plant seeds contain the adequate element mix for near-optimal growth during the first period of time after germination^7^. Hence, seed elemental stoichiometry is expected to be similar to an actively growing plant when nutrient supply and growing conditions are near-optimal. Autumn-sown annual crops, like winter wheat, are subject to conditions unfavorable for growth during the winter. Thus, for such crops, we expect similar element stoichiometry in actively growing plant tissues and seeds (both initial and harvested seed), but a different stoichiometry after the winter, i.e. at the onset of rapid growth in early spring. The early-spring element concentrations will be determined by the balance between element availabilities in combination with plant uptake capacity and growth during the period between the initial seedling stage in autumn and the start of the active growth in spring. The winter conditions during this period expose plants to situations deviating markedly from steady-state conditions (i.e. constant nutrient concentrations over time^11^), which may result in greatly enhanced tissue concentrations of elements early in the growing season as long as the uptake of these elements occurs prior to the onset of substantial growth^8^. When projected into a whole life cycle-perspective “from seed to seed” of a winter-annual crop like winter wheat, we may expect to see enhanced element concentrations of vegetative plant tissues (compared to seed) early in the growing season, but thereafter declining concentrations during the growth period down to or even below the corresponding concentrations in seeds; depending on how essential each element is for the maintenance of important growth functions. In addition, the temporal dynamics of element concentrations should also be affected by weather^4^,^12^.

Many agricultural crops are frequently grown in continuous monoculture without a break crop, even though they might provide lower yields when grown in monoculture than when grown after another species (i.e., in a rotation with a different preceding crop)^13^,^14^. For example for wheat, preceding crops used as elements in crop rotations may affect wheat growth and yield partly through their influences on the soil N and/or other nutrients^14^. If, in a crop rotation, the preceding crop effect is mediated by soil nutrients, a corresponding change in the wheat nutrient concentrations and/or stoichiometry might be already apparent in the short term (e.g. within one or two years); i.e., well before any possible crop rotation effects on wheat yields are likely to be detected. A short-term effect of preceding crop on element stoichiometry of the main crop grown in a crop rotation could provide an interesting tool for the early detection of the nutrient-related effects of crop rotations on crop yields that frequently become apparent only in the long term. We are not aware of any studies investigating short-term preceding crop effects by means of element concentrations and stoichiometry including various nutrients beyond N and P.

Wheat is one of the most commonly grown arable crops and the dominating plant in many areas of the world. Using winter wheat as a model, the overall objective was to investigate element concentration pattern as affected by crop developmental stage (in a seed-to-seed perspective) and weather. We also aimed at evaluating whether element stoichiometry reflects the type of preceding crop in the short term of one to two years. We explored the following main hypotheses: (1) Element concentration pattern varies across developmental stages, with the largest deviation from seed stoichiometry in early spring (before the initiation of main growth and nutrient uptake), when element concentrations should be much higher than in seeds but thereafter decline to or below the corresponding element concentrations in seed; (2) Element concentration pattern reflects weather; (3) Among all the elements, N has the strongest influence on the element concentration pattern. The aims and hypotheses were addressed by assessing temporal trajectories of element concentration pattern across the life cycle of winter wheat, grown in the field over two years with contrasting weather (Fig. 1), and as affected by different preceding crops (Table 1).

## Results

**Figure 1:**
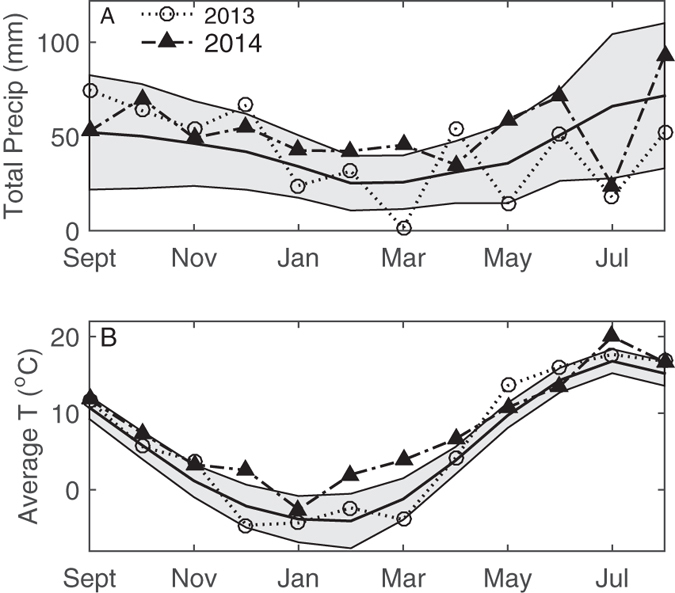
Growing conditions during the experimental period. (**A**) total monthly precipitation and (**B**) average air temperature. ‘2013’ refers to the period September 2012-August 2013, ‘2014’ to September 2013-August 2014. Long-term (1896–2014) average (solid black line) and average ± standard deviation (shaded areas) are plotted as a term of comparison. All the data were collected at the Ultuna climate station near Uppsala, situated 3 km south-west from the experimental site at Säby.

Inter-annual differences in weather conditions (e.g. dry in 2013 and wet in 2014; Fig. 1) were reflected by great variation in biomass growth and grain yield between the two years (Table 2). Within each year, grain yield was similar in the monoculture and the different preceding crops (Table 2).

**Figure 2:**
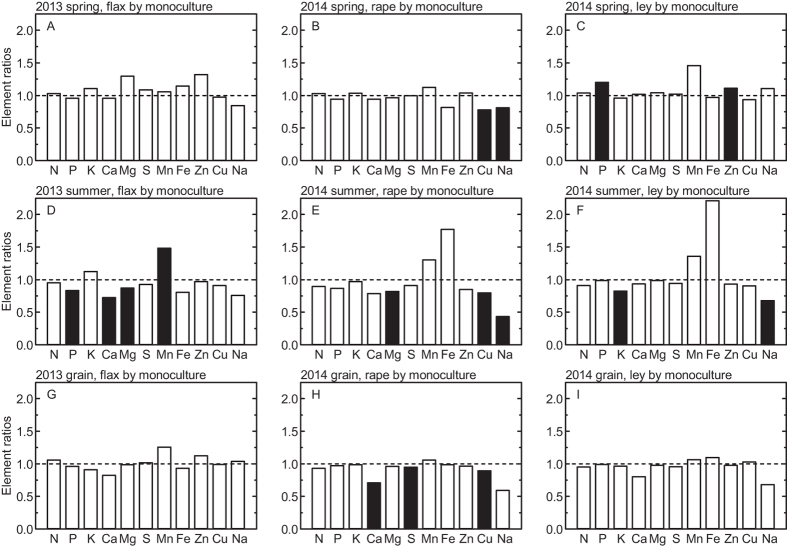
Element concentration ratios (means in preceding crop divided by means in monoculture) in winter wheat above ground biomass (spring at BBCH 31, (**A–C**) summer at BBCH 61, (**D–F**) and grain yield (**G–I**) field-grown in Central Sweden during two growing seasons (2013 and 2014). Closed bars indicate significant differences (*P* < 0.05) in element concentrations when comparing the respective preceding crop (flax, rape or ley) and monoculture.

**Figure 3:**
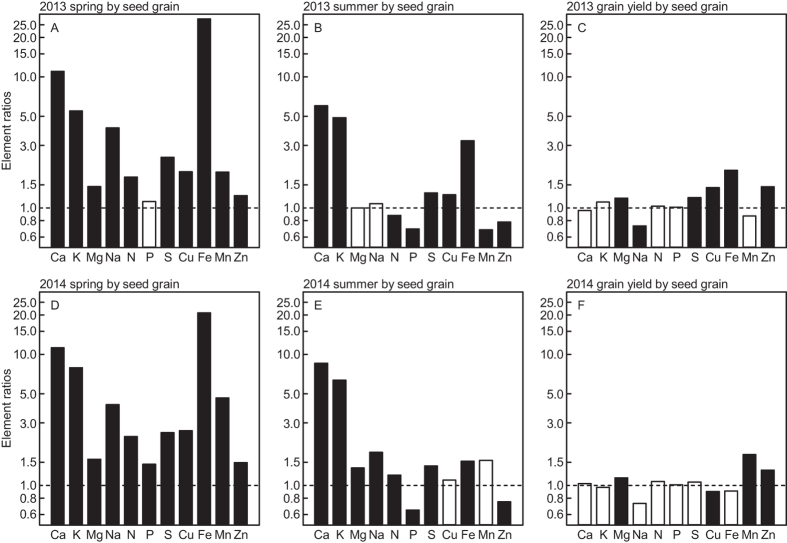
Element concentration ratios (means at different developmental stages by seed grain). Means across different preceding crops in spring (**A,D**) summer (**B,E**) and grain yield (**C,F**) in winter wheat (spring at BBCH 31, summer at BBCH 61) field-grown in monoculture in Central Sweden during two growing seasons (2013, **A–C**; and 2014, **D–F**). Closed bars indicate significant differences (*P* < 0.05) in element concentrations when comparing the spring, summer and grain yield, respectively, with the seed grain. Note: y-axis is in log_e_ scale.

**Figure 4:**
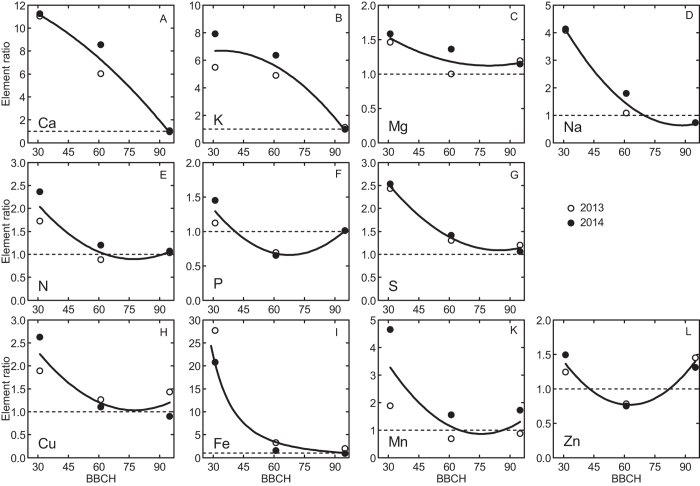
Element concentration ratios (means at different developmental stages divided by seed grain) as functions of developmental stage (BBCH) in winter wheat field-grown in monoculture in Central Sweden during the two growing seasons (2013 open symbols; and 2014 closed symbols; for graphical clarity, only the means for each developmental stage and year are reported). Quadratic functions were fitted to most of the data, except Fe for which a power function was fitted. Note the different y-axis scales.

The concentrations of many elements were significantly affected by year; also preceding crop treatment resulted in significant variation in the concentrations of some elements but not others (Table 3). However, no consistent patterns of element concentration effects caused by year or preceding crop were apparent (Table 3; supplemental Figs S1–S3). Element ratios of the concentrations in the preceding crop treatment divided by the corresponding value for monoculture showed several significant effects (black bars in Fig. 2), with either higher (P, Mn, Zn) or lower concentrations (P, K, Ca, Mg, S, Cu, Na) for some preceding crop but not for others. No consistent pattern was apparent regarding the effect of preceding crop (Fig. 2). Element ratios calculated as the concentrations in plants or grain at different developmental stages divided by the corresponding concentrations in the seed grain (sown seed) indicated for nearly all elements significantly higher concentrations in the plants at the start of stem elongation in spring compared to seed grain. More moderate differences emerged at anthesis and mostly small variation between harvested grain and seed grain (Fig. 3). The pattern was similar between the two years. The element concentration ratios of two elements (Ca, K) greatly decreased across the whole vegetation period; the one of seven elements (N, S, Mn, Mg, Fe, Cu, Na) decreased mostly during spring but were more stable during summer; whereas the element concentration ratios of the two further elements (P, Zn) decreased during spring and increased during summer (Fig. 4).

**Figure 5:**
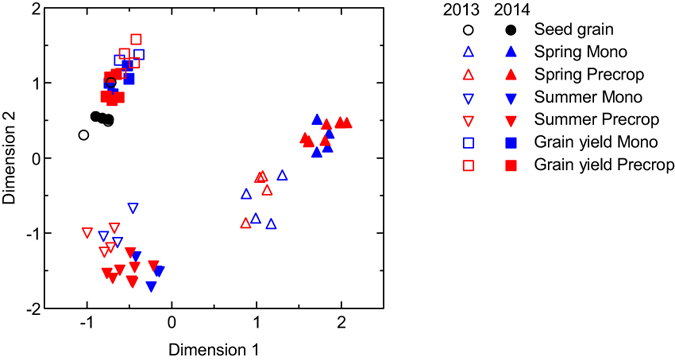
Grouping of samples according to Principal Components Analysis (PCA). Samples are replicates of element concentrations in winter wheat at different developmental stages, i.e. seed grain, above ground plant biomass in spring (BBCH 31), above ground plant biomass in summer (BBCH 61), and grain yield. The wheat was field-grown in Central Sweden during two growing seasons (2013, open symbols; and 2014, closed symbols) and with different preceding crops (wheat monoculture, blue symbols; non-wheat preceding crop, red symbols). Eigenvalues 7.78 for dimension 1 (explanatory power 71%), and 1.76 for dimension 2 (explanatory power 16%).

The grouping of all samples in a Principal Components Analysis (PCA) clearly separated three groups of samples: i) plant samples from the start of stem elongation in spring regardless of the preceding crop, with a clear separation of the two years; ii) plant samples taken at anthesis regardless of the preceding crop, with a less clear separation of the samples from the two years; and iii) seed grain and harvested grain samples regardless of the preceding crop and year (Fig. 5). The pattern indicates a strong effect of time on element concentrations, and a strong year effect on element concentrations mainly during spring. Correlation coefficients for the PCA dimension 1 were highest for N, followed by S, Cu and Fe; P and Zn showed high component loadings for the PCA dimension 2 (Table 3). This means that N explained most of the variation (i.e. 96%) along PCA dimension 1, which also explained 4.4 times more of the total variation in the data set compared to PCA dimension 2 (see eigenvalues, Fig. 5). Keeping in mind the much greater eigenvalue of PCA dimension 1, it is notable that plant samples at anthesis were separated from grain samples only in PCA dimension 2 (Fig. 5). Among the supplemental variables (year, time, preceding crop), the developmental stage (time) showed high component loading in the PCA dimension 2, whereas year and preceding crop showed low component loadings (Table 3). Elements can be grouped in the concentration space in the sense that nutrients in the same group are changing in concert. The emerging clusters confirm the element groups that we identified previously when comparing the temporal trajectories of element concentrations (Fig. 4). Thus, K and Ca had high component loadings in PCA dimension 1 and low loadings in dimension 2 (Table 3); a large group of elements (N, S, Mn, Mg, Fe, Cu, Na) showed high component loadings in component 1 and medium loadings in dimension 2; and two elements (P, Zn) had high component loadings in both dimensions.

## Discussion

The paper reports for the first time the element stoichiometry and temporal trajectories of the concentrations of 11 nutrient elements across the whole life cycle of a plant. We found considerable variation in element concentrations and stoichiometry between years (differing in environmental conditions) and crop developmental stages, similar results were also reported elsewhere and for various plants^4^,^5^,^8^,^9^,^10^. In contrast to most other studies of element stoichiometry in plants, we included several nutrient elements beyond N. Further, we considered the entire plant life history “from seed to seed”. Accordingly, we assumed that plant seeds contain an adequate nutrient mix to support vegetative growth for a limited period of time after germination^7^, and therefore argued that seed element stoichiometry reflects an optimum nutrient mix for growth in the environment to which the plant is adapted. Optimum nutrient mix for growth can also be assumed in vegetative tissues of actively growing plants when nutrient supply and growing conditions are near to optimal^5^; a situation frequently desired in modern agriculture of e.g. winter wheat during the period between start of stem elongation and anthesis. Nevertheless, autumn-sown annual crops, like winter wheat, are subject to stressful conditions during the winter. Hence their stoichiometry at the beginning of stem elongation may be different from the optimal one. Based on this rationale, we focused here on the temporal trajectories of element stoichiometry across the whole life cycle of a conventionally fertilized, field-grown winter wheat in a cool-temperate climate, considering both reproductive units (seeds) and growing plants as comparable functional units. We explored hypotheses regarding the effects of developmental stage and environment (year-to-year variation in weather) on element concentration pattern. On top of the more theoretical considerations mentioned above, this analysis is interesting also in a more practical perspective, as it provides a test for the common assumption that temporal dynamics in plant nutrient concentrations are largely reflected by the pattern in N concentrations.

We expected vegetative tissue element concentrations to be generally higher than in the seed grain early in the growing season directly after winter, partly because some nutrient uptake may occur during periods in late fall and winter without concomitant growth, and partly because nutrient uptake rate after winter might accelerate more rapidly than biomass growth. In line with our hypothesis, we mostly found significantly higher element concentrations at the start of stem elongation in spring, except for P in year 1 of the study (Fig. 3A,C). Indeed, periods early in the growing season with enhanced nutrient uptake rates not matched by proportional growth have been reported for many crops^8^. In our study, the concentrations of most elements decreased from early spring (i.e. start of stem elongation) to the end of the growing season; seed and harvested grain had similar concentrations (Fig. 4), which was also expected. Notable exceptions from this seasonal pattern were the concentrations of P and Zn, which dropped at anthesis considerably below the corresponding seed concentrations. This decline in P and Zn concentrations might reflect the generally slow uptake of these elements and its dependence on soil microbes and/or mycorrhiza active also during the period after termination of most vegetative growth at anthesis^15^. The greatly enhanced concentrations of most elements at the start of the elongation stage, in concert with the great variability across individual elements especially at this developmental stage, resulted in a clear separation of the spring samples in the PCA (Fig. 5). These results support our first hypothesis and indicate that element concentration pattern varies during the growing season, with a maximum deviation from seed stoichiometry in early spring.

This study included two years with contrasting weather, i.e. dry (2013) and humid (2014) early growing season (Fig. 1), which resulted in large (by factor 2) grain yield differences between the two years. The inter-annual variability in weather was expected to affect element stoichiometry as reported by others^4^,^12^. The concentrations of all elements were significantly influenced by year at some developmental stage(s). Interestingly, the significant effects of year on the element concentrations in grain were seen for all micronutrients (Mn, Fe, Zn, Cu), but not for the other elements studied here (Table 3). Micronutrient concentrations in wheat grain are known to be affected by the soil bioavailability of macronutrients such as N^16^, but N fertilization was similar in the two years of our study. A two year period is too short to draw any general conclusions on year-to-year variation in element concentrations. However, our observations on the inter-annual variation in grain micronutrient concentrations are generally in line with other results from wheat^17^. The small year-to-year variation in grain nutrient concentrations of most elements (except for the micronutrients) despite a large difference in grain yield indicates that these plants have been able to largely control uptake and re-translocation of these essential elements to the grain^18^. Plant samples at anthesis and grain samples were not separated in the dimension 1 of the PCA (Fig. 5). This result is suggestive that the element stoichiometry in grain reflects an optimum nutrient mix for growth in a similar way as in a vegetative plant grown under near-optimal conditions for growth. The relatively small effect of year on the total variation in element concentrations in this study is reflected by the low component loading values for this variable in the PCA (Table 3), although the grouping of samples according to PCA revealed some separation of the 2013 and 2014 samples especially in spring (Fig. 5).

Wheat culture in crop rotations with different preceding crops often generates higher yields compared to wheat monoculture, and the action of soil nutrients has been suggested to be involved in explaining the higher wheat yields observed in crop rotations^13^,^14^. On the one hand, any effects of crop rotations on the nutrient stoichiometry of the main crop (here wheat), if mediated by soil nutrients, would be most likely to be seen in the long term, i.e., after multiple years. On the other hand, preceding crop effects on crop yields have been reported also in the shorter term^19^. Further, any evidence of a short-term effect of preceding crop on element stoichiometry of the main crop grown in a crop rotation could provide an interesting possibility for developing a tool for the early detection of the nutrient-related effects of crop rotations on crop yields that, if detectable at all, often become apparent only in the long term. Therefore, we tested whether the type of preceding crop (non-wheat preceding crop *vs.* wheat monoculture) could be reflected in element concentration pattern of the wheat crop already in the short term of one to two years. We found significant effects of preceding crop type for most elements (except N, S and Fe) in some year and/or developmental stage, but no systematic pattern emerged (Table 3, Fig. 2) and different preceding crops were not separately grouped in the PCA (Fig. 5). These results therefore cannot provide any evidence for a short-term preceding crop effect on yields mediated by soil nutrients, which has been suggested as one of the potential mechanisms explaining the higher wheat yields frequently achieved in crop rotations as opposed to monocultures^14^. We underline that the design of our study does not allow any conclusion on possible long-term effects of crop rotations on the nutrient stoichiometry and yield of wheat grown as the main crop in a crop rotation. Our long-term field trial accommodates so far only a short history of different crop rotations (assessments here were done three and four years after trial establishment), and we have hitherto not seen any clear effect of different crop rotations on wheat yields in this trial (e.g. Table 2). The fact that we did not find any clear evidence here for a nutrient-related mechanism explaining part of a short-term preceding crop effect on wheat yields means either that, in this particular trial, wheat yields are not influenced by the preceding crops; that potential future effects of different crop rotations on yields will not be related to nutrients; or that any nutrient-related effect of the investigated crop rotations on wheat yields will become apparent only in the long term (several years). To disentangle these possibilities, we plan to follow up this study in the future. In this context, it needs also to be kept in mind that preceding crop effects on wheat yields might vary between years, depending on climate and weather conditions^19^. In addition, it should be born in mind that any specific effects of preceding crops on the nutrient relationships and yield of a main crop (here wheat) grown as an element of commonly used crop rotations (e.g., the ones tested here^20^) can only be assessed in the short term, because these crop rotations contain only one or a few years of non-wheat crops preceding the main crop. Based on the results of this study, we found some evidence supporting our second hypothesis, because the different climate in the two years was reflected in the nutrient concentration pattern (particularly in spring, where also weather differences between years were greatest). The results are less encouraging with respect to the development of a tool for the early detection of the nutrient-related effects of crop rotations on crop yields, because the influence of the preceding crop on nutrient concentration pattern was weak in the short term (one to two years) investigated here.

As nitrogen is among the most important elements limiting plant growth and crop production, we expected N to exert a strong influence on the observed concentration pattern for all elements. Maximum component loading for N in the PCA (Table 3) is convincing evidence for the prominent role of N in explaining (or representing) the element concentration pattern across the whole dataset, and therefore confirms our third hypothesis. This means that the variation in element concentration patterns in this study was closely mirrored by the pattern in N concentration, which supports the corresponding common assumption made in many nutrient research studies. However, different elements had different temporal trajectories across the growing seasons and also responded differently to the type of preceding crop. This is suggestive of the individual transport mechanisms and functional roles for each of these essential elements^6^,^21^. The observed temporal trajectories of concentrations and the PCA results suggest that nutrients can be grouped stoichiometrically, i.e., such that the concentrations of elements in the same group are changing in concert (Figs 4 and 5). Thus, a group of two elements (K and Ca) had high component loadings in PCA dimension 1 and low loadings in dimension 2 (Table 3). The elements K and Ca are similar in size, valency and ion charge and were here characterized by very high concentrations (and accumulation rates; data not shown) at the start of stem elongation in spring followed by strongly and progressively declining concentrations throughout the growing season (Fig. 4). The second and largest group consists of seven elements (N, S, Mn, Mg, Fe, Cu and Na) with similar temporal concentration trajectories (i.e. quickly decreasing during spring and stable thereafter) despite great differences in concentration ranges within the group. A third group is formed by two elements (P and Zn) with high PCA component loadings in both dimensions. The P and Zn were the only elements in this study for which the concentrations at anthesis dropped considerably below the corresponding seed concentrations – a pattern that could be related to the generally slow uptake rates and/or the strong dependence on soil microbes and/or mycorrhiza for these nutrients, as discussed elsewhere^15^. With reference to our third hypothesis, our data clearly confirm the prominent role of N in explaining the element concentration pattern. However, including additional nutrients so that each of the three groups identified here is represented (e.g. N, P, K) would provide a more complete picture of nutrient research issues related to the ones investigated here.

Apart from the developmental stage and weather, the nutrient concentration pattern in plants is influenced also by the nutrient bioavailability in the soil^1^,^10^. However, the nutrient concentrations in plants often reflect nutrient uptake conditions more than nutrient availabilities in the soil, and the relationships between the concentrations of individual nutrient elements in the plant and soil are not linear^1^. Because here we considered only one soil exposed to common-practice nutrient fertilization in Sweden, we cannot rule out that other soils would have produced slightly different patterns compared to the results reported here. Similarly, the consideration of wheat varieties other than ‘Olivin’, which is commonly grown in Sweden, could result in patterns different from those reported here. Nevertheless, our results were obtained from a commonly used wheat variety grown in a representative field site for cool-temperate Sweden throughout two consecutive years. Thus, we believe that the main directions of element stoichiometry pattern identified here are valid also in other contexts.

In conclusion, the seed-to-seed approach applied here provided novel insights into the temporal dynamics of nutrient concentration pattern. We identified three groups of elements with concentrations changing in concert. We found strong influence of developmental stage on element concentration pattern, with greatest variation between concentrations in grain and vegetative plant at the start of stem elongation in spring (cf. hypothesis 1). Inter-annual differences in weather were reflected in nutrient stoichiometry (cf. hypothesis 2). In the short term (one to two years), preceding crop was only weakly reflected by the element concentration pattern of the main crop (wheat) in a crop rotation. The variation in nutrient concentration pattern was closely mirrored by the pattern in N concentration (cf. hypothesis 3).

## Methods

### Study site

The study was carried out during the 2013 and 2014 growing seasons in a long-term field experiment at Säby (R4-0009), 5 km south of Uppsala, Sweden (59° 45′ N, 17° 42′ E)^20^. The soil at the experimental site is a Cambisol formed in postglacial sediments. The soil texture is a silty clay (British standards institution; 15% clay, 55% silt, 30% sand) with an organic matter content of 4% and pH (H_2_O) 6.1. The mean total carbon (C) and N concentrations were 28.1 and 2.5 g kg^−1^, respectively, at 0–60 cm soil depth; the corresponding pseudo-total concentrations (ammonium lactate extractable) of P, K, Mg, Ca, Al, Fe were 0.1, 0.1, 0.1, 2.2, 0.3, 0.4 g kg^−1^, respectively (unpublished data from soil sampling in November 2010 based on dry soil samples milled and sieved at 2 mm before analysis). The soil C concentrations were measured using dry combustion and infrared gas analysis (LECO). Climate in Uppsala is boreal-temperate and the growing season normally lasts from April to October. Spring 2013 was drier than normal with mean temperature similar to or lower than long-term mean, whereas spring 2014 was wetter and warmer than the long-term mean (Fig. 1).

### Experimental design and crop management

The Säby long-term field experiment^20^ was established in 2010 with a randomized complete block design with four replicates and an individual plot size of 420 m^2^. Sampling for this study was conducted in 6-years crop rotations and a monoculture of winter wheat (*Triticum aestivum* cv. ‘Olivin’). In 2013, wheat was sampled in the monoculture and in one rotation with flax as the preceding crop; in 2014, wheat was sampled in the monoculture and two crop rotations, i.e. with winter rape and grassland ley as preceding crops (Table 1). Further details about the crop rotations are reported elsewhere^20^. Conventional tillage system including ploughing and disk harrowing was performed in the monoculture and crop rotations studied here. Wheat seeds were sown on 10^th^ October 2012 (prior to the 2013 growing season) and 13^th^ September 2013 (prior to the 2014 growing season). Seed rate was 540 seeds m^−2^, as common in the region. Wheat was fertilized with P and K before autumn sowing (2.6 and 5.0 g m^−2^, respectively) and with 12.0 g N m^−2^ (corresponding to 120 kg ha^−1^) in the spring. This amount of N is near the recommended dose for winter wheat on fertile soils in this area. The preceding crop flax was sown on 16 May 2011 and received 2.1 and 4.0 g m^−2^ P and K, respectively, in autumn of the same year; no N fertilizer was applied to the flax plots. The flax was harvested in October 2012 and winter wheat was sown immediately thereafter. The preceding crop winter rape was sown in August 2012 and received 3.1 g m^−2^ of P and 6.0 g m^−2^ of K at sowing, and 11.0 g N m^−2^ in the following spring. The rapeseed was harvested in September 2013, followed by winter wheat seed bed preparation and sowing. The preceding crop grassland ley was under-sown in barley in autumn 2011 and consisted of a mixture of 35% timothy (*Phleum pretense* L., cv Gate City), 35% perennial ryegrass (*Lolium perenne* L. cv Birger), 25% meadow fescue (*Festuca pratensis* L. cv Sigmund), 10% red clover (*Trifolium pretense* L. cv. Ares), 10% alfalfa (*Medicago sativa* L. cv Nexus) and 5% white clover (*Trifolium repens* L. cv Ramona). The plots received 6.1 g N m^−2^, 2.1 g P m^−2^ and 4.0 g K m^−2^ in October 2011. Additional 5.5 g N m^−2^, 2.5 g P m^−2^ and 9.0 g K m^−2^ were applied to the plots in spring 2012 and 2013. Barley was harvested in August 2011 and the under-sown grassland ley was cut in October 2012. In 2013, the ley was cut twice (15^th^ June and 24^th^ July) before it was plowed under in August 2013 followed by winter wheat seed bed preparation and sowing, as customary in the region.

### Sampling

Sampling of aboveground plant parts was done between September 2012 and August 2014 within a 36-m^2^ subplot in the southern corners of the individual plots^20^. Samples of winter wheat seed grain (autumn 2012 and 2013) were dried and stored for later element analysis. The aboveground biomass (taken from the ground level) from randomly selected 0.32-m^2^ areas of each subplot was sampled when approx. 50% of the wheat plants within the individual plots were at the following developmental stages (BBCH^22^): three leaf stage (BBCH 13; around 12^th^ October in 2012 and 2013), at start of stem elongation (BBCH 31; 13^th^ May in 2013 and 14^th^ March in 2014), at anthesis (BBCH 61; around 13^th^ June in 2013 and 2014), and at the end of maturity (BBCH 99; 13^th^ August in 2013 and 2014). At the last harvest at BBCH 99, winter wheat straw and grain were harvested separately; grain threshing was performed manually. All samples were oven-dried at 70 °C for 36 h, weighed and ground in a stainless steel grinder to pass a 1-mm mesh before nutrient element analysis. N concentration was analyzed on a LECO CNS/2000 analyzer using a standard method (SS-ISO13878). The contents of P, K, Ca, Mg, S, Mn, Fe, Zn, Cu and Na were extracted using 32.5% Nitric Acid on a heat block and concentrations were determined using ICP-AES technique (Spectro Blue FMS 26, Spectro Analytical Instruments, Kleve, Germany) by applying internal standardization protocols.

### Statistical analysis

The SPSS version 22 procedure General Linear Model was used for calculating probabilities of significant differences in element concentrations for effects of preceding crop, year and developmental stage. In 2013, the preceding crops were monoculture (i.e. wheat was here statistically treated as a preceding crop) and flax. In 2014, the preceding crops were monoculture, winter rape and ley. Effects of Year (2013 and 2014) were calculated using data only from the monoculture. The SPSS version 22 procedure CATPCA was used to group the samples according to element concentrations pattern in Principal Components Analysis (PCA) and relate the grouping to the supplementary variables year (representing weather), time (representing crop developmental stage), and preceding crop. Element concentrations were defined as numeric (continuous) variables, and supplementary variables were defined categorical.

## Additional Information

**How to cite this article**: Weih, M. *et al*. Nutrient stoichiometry in winter wheat: Element concentration pattern reflects developmental stage and weather. *Sci. Rep.* **6**, 35958; doi: 10.1038/srep35958 (2016).

## Supplementary Material

Supplementary Information

## Figures and Tables

**Table 1:** Schematic outline of the crop sequence arrangements sampled in the Säby long-term experiment near Uppsala, Sweden (R4–0009)^20^.

*Year*	2011			2012			2013			2014	
*Time of year*^1^	*I*	*II*	*III*	*I*	*II*	*III*	*I*	*II*	*III*	*I*	*II*
*Wheat monocult.*	W	W	W	W	W	**W**	**W**	**W**	**W**	**W**	**W**
*C*_*P*_ *Flax*	W	W	F	F	F	**W**	**W**	**W**	R	R	R
*C*_*P*_ *Rapeseed*	F	F	W	W	W	R	R	R	**W**	**W**	**W**
*C*_*P*_ *Ley*	B	B	L^2^	L	L	L	L	L	**W**	**W**	**W**

Sampling of plant material was done only in wheat (W, bold dark letters). The light letters indicate the here not sampled crops of the crop sequences, including the preceding crops (*C*_*P*_) flax (F), rapeseed (R), ley (L) and additionally also barley (B) in the *C*_*P*_ Ley sequence. Winter crops were sown in the autumn preceding the year in which they were harvested.

^1^*I* Jan – Apr, *II* May – Aug, *III* Sep – Dec.

^2^Under-sown in barley (B).

**Table 2:** Means and standard deviations (in parentheses) of above ground biomass (*B*) during the main growth period and grain yield of winter wheat, grown in monoculture (Mono) and with various preceding crops (Flax, Rape, Ley) in two years (2013 and 2014 growing seasons) in a long-term field experiment.

Trait*	Year 1		Year 2		
	Mono	Flax	Mono	Rape	Ley
*B* at BBCH 31 (g m^−2^)	24.4 (12.6)	25.2 (5.8)	38.1 (13.8)	37.0 (3.3)	38.8 (10.0)
*B* at BBCH 61 (g m^−2^)	144 (53)	276 (17)	919 (55)	899 (50)	1008 (168)
*R*_*G*_ (week^−1^)	0.26 (0.13)	0.35 (0.04)	0.35 (0.04)	0.35 (0.01)	0.36 (0.04)
Grain yield (g m^−2^)	323 (113)	299 (117)	656 (72)	684 (25)	721 (84)

*R*_*G*_ relative growth rate between start of stem elongation (BBCH 31) and anthesis (BBCH 61).

*P values for significant differences between preceding crops and years were mostly <0.05, except for <0.01 in the cases of treatment differences for *B* at BBCH 61 (year 1), and year differences (only data from monoculture considered) for grain yield and *B* at BBCH 61.

**Table 3:** General Linear Model and Principal Components Analysis (PCA) results for winter wheat field-grown in Central Sweden.

Element	Plants at BBCH 31			Plants at BBCH 61			Grain yield			PCA	
	C_P_ 2013	C_P_ 2014	Year	C_P_ 2013	C_P_ 2014	Year	C_P_ 2013	C_P_ 2014	Year	Dim. 1	Dim. 2
N	—	—	**	—	—	—	—	—	—	0.981	0.172
P	—	**	*	*	—	—	—	—	—	0.762	0.562
K	—	*	**	—	*	**	—	—	—	0.705	−0.652
Ca	—	—	*	**	—	—	—	*	—	0.771	−0.595
Mg	—	—	—	*	**	**	—	—	—	0.864	−0.043
S	—	—	**	—	—	*	—	—	—	0.960	−0.064
Mn	—	—	—	*	—	*	—	—	*	0.761	0.071
Fe	—	—	—	—	—	**	—	—	**	0.931	−0.029
Zn	—	*	—	—	—	—	—	—	*	0.556	0.771
Cu	—	*	**	—	*	—	—	**	**	0.937	0.102
Na	—	**	—	—	**	*	—	—	—	0.917	−0.138
Year§										0.146	−0.003
Time§										−0.393	0.716
C_p_§										0.132	−0.057

Probability values (- *P* > 0.05, * 0.01 < *P* < 0.05, **P < 0.01) in element concentrations for effects of preceding crop (C_P_ - monoculture *vs.* different preceding crops) of plants at two developmental stages (BBCH) and grain yield in two years (2013 and 2014); and Year (here considering only data from the monoculture). Correlations between the first two principal components (Dimensions 1 and 2) and the original variables, according to PCA presented in Fig. 5; components are element concentrations and the supplementary variables year (representing weather), time (representing crop developmental stage), and C_p_.

^§^Supplemental categorical variables in PCA.
