# Supplementary material for: Nutrient stoichiometry in winter wheat: Element concentration pattern reflects developmental stage and weather
Source: Sci Rep. 2016 Oct 24;6:35958. doi: 10.1038/srep35958 (PMC5075900; doi:10.1038/srep35958)
Supplement: Supplementary Information [file srep35958-s1.pdf]

Scientific Reports **Supporting Information**

Article title: **Nutrient stoichiometry in winter wheat: Element concentration pattern reflects developmental stage and weather**
Authors: M. Weih, F. Pourazari, G. Vico

The following Supporting Information is available for this article:

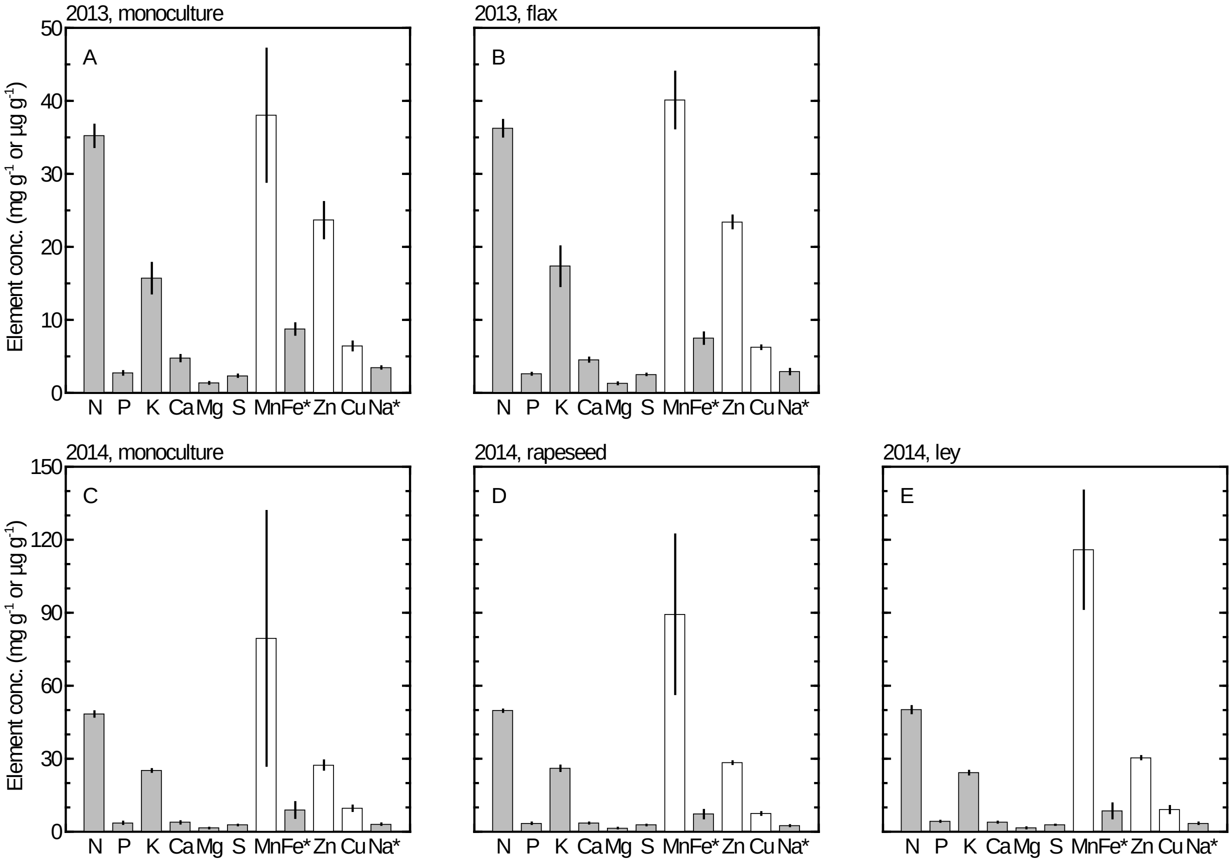

Figure S1. Means (bars) and standard deviations (whiskers) of element concentrations in **above ground biomass** of winter wheat at **BBCH 31** (start of stem elongation in spring) grown in monoculture and with various preceding crops (flax, rape, ley) in 2013 (A, B) and 2014 (C to E) in a long-term field experiment (Säby R4-0009) in Central Sweden. Units are mg g^{-1} (closed bars) for most of the elements except Mn, Zn and Cu, for which the unit is µg g^{-1}(open bars); * for Fe and Na means mg g^{-1} x 10. Note the difference in the y-axis scale between top and bottom rows.

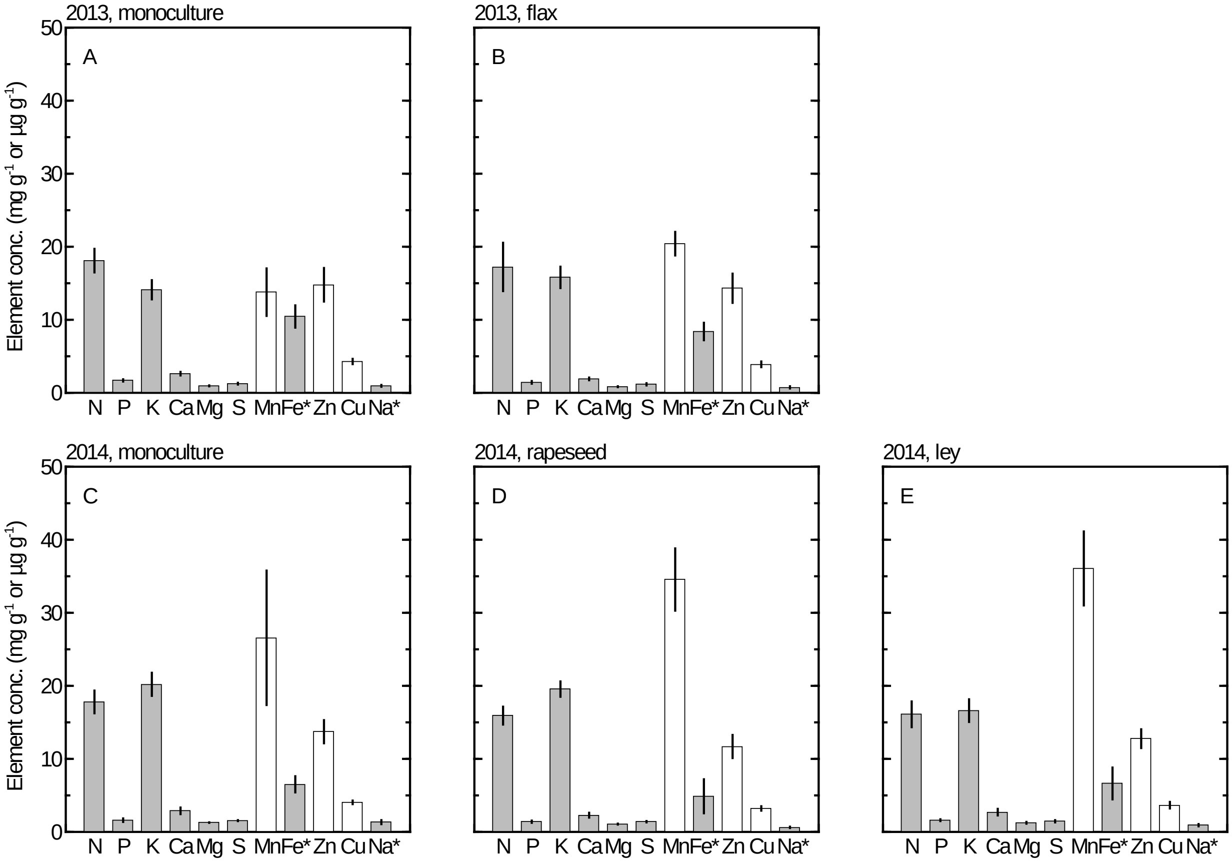

Figure S2. Means (bars) and standard deviations (whiskers) of element concentrations in **above ground biomass** of winter wheat at **BBCH 61** (anthesis) grown in monoculture and with various preceding crops (flax, rape, ley) in 2013 (A, B) and 2014 (C to E) in a long-term field experiment (Säby R4-0009) in Central Sweden. Units are mg g^{-1} (closed bars) for most of the elements except Mn, Zn and Cu, for which the unit is µg g^{-1}(open bars); * for Fe and Na means mg g^{-1} x 10.

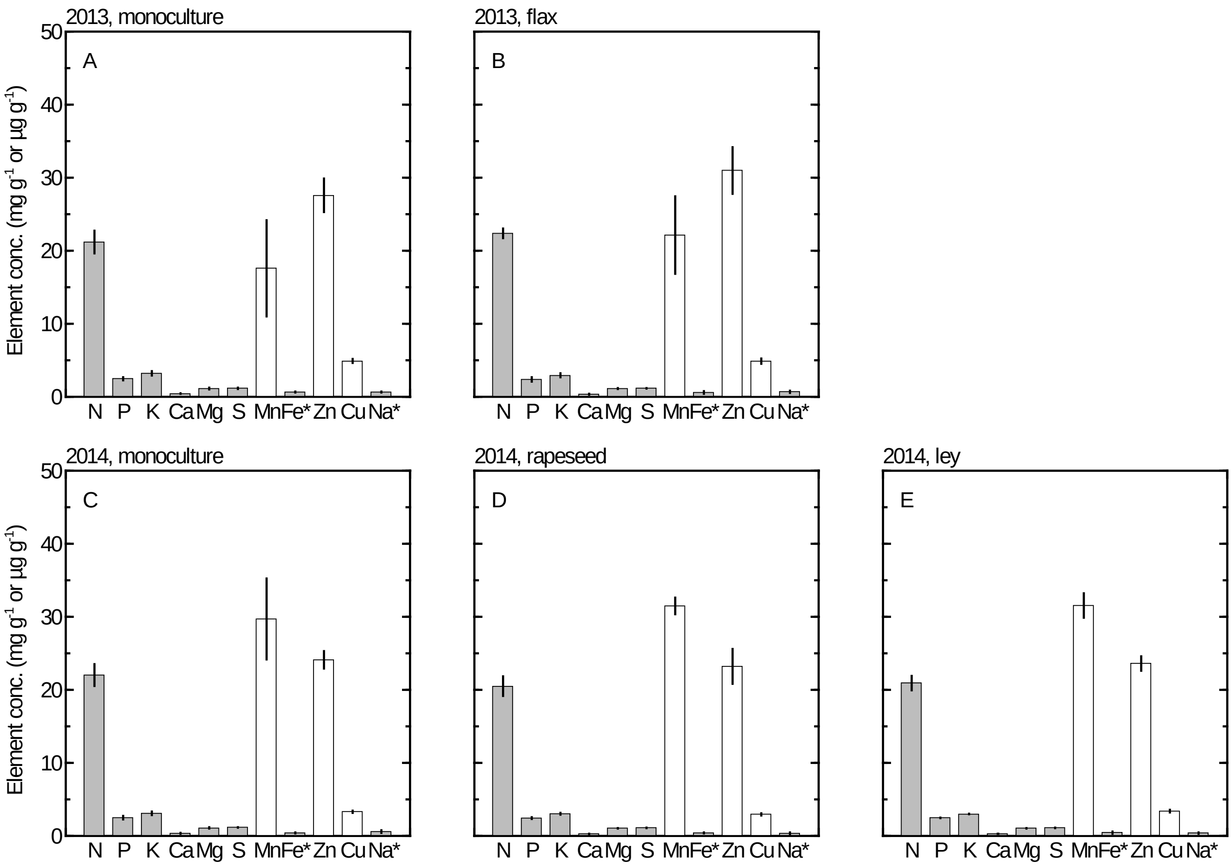

Figure S3. Means (bars) and standard deviations (whiskers) of element concentrations in **grain** yield of winter wheat grown in monoculture and with various preceding crops (flax, rape, ley) in 2013 (A, B) and 2014 (C to E) in a long-term field experiment (Säby R4-0009) in Central Sweden. Units are mg g^{-1} (closed bars) for most of the elements except Mn, Zn and Cu, for which the unit is µg g^{-1}(open bars); * for Fe and Na means mg g^{-1} x 10.
